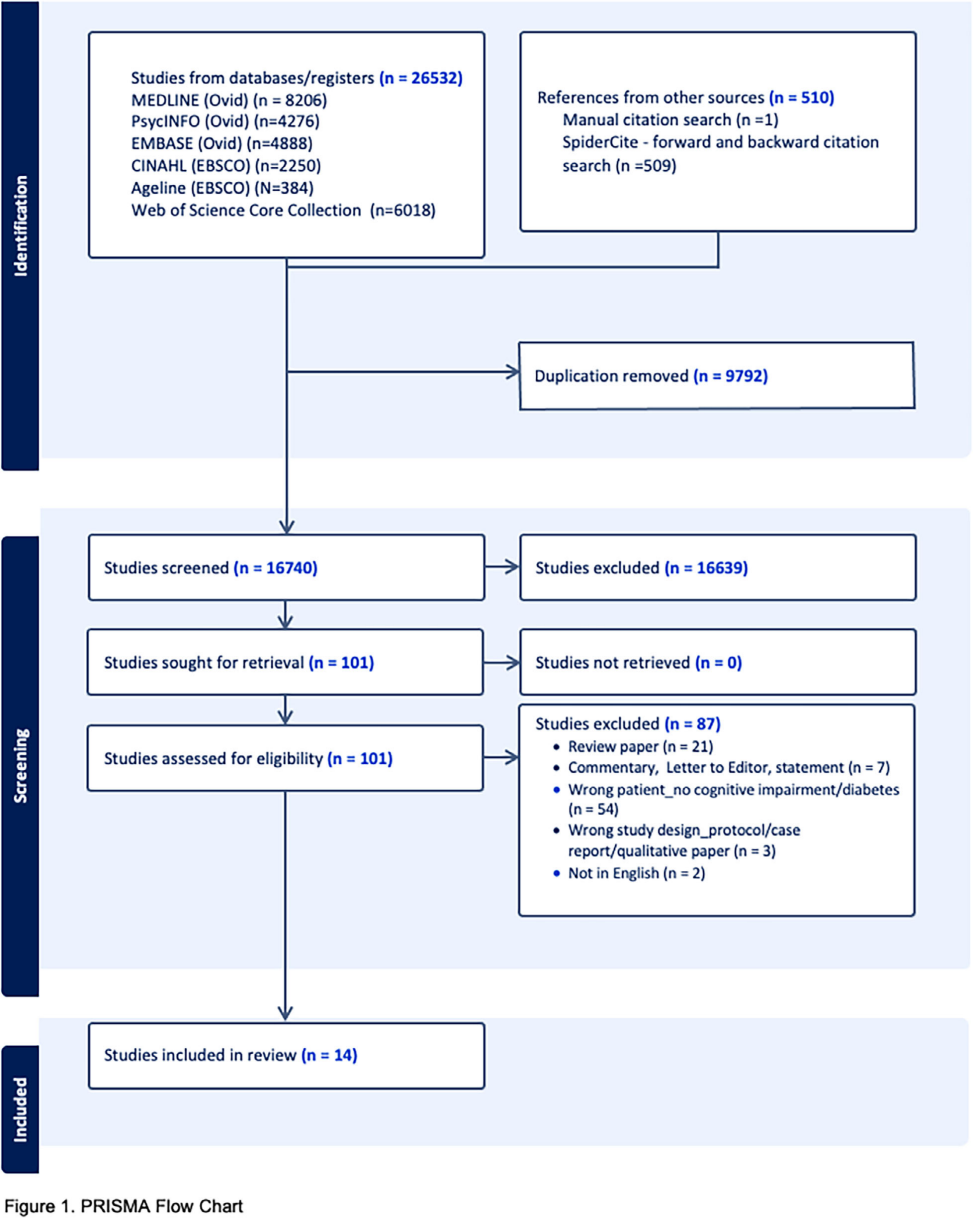# A Scoping Review of Non‐pharmacological Interventions for Adults with Cognitive Impairment and Diabetes

**DOI:** 10.1002/alz.088488

**Published:** 2025-01-09

**Authors:** Yaguang Zheng, Brynne Campbell Rice, Judith Wylie‐Rosett, Bei Wu

**Affiliations:** ^1^ New York University, New York, NY USA; ^2^ Albert Einstein College of Medicine, Bronx, NY USA; ^3^ NYU Aging Incubator, New York, NY USA

## Abstract

**Background:**

Adults with comorbidity of cognitive impairment and diabetes is common (19.9%‐45.0%) and increasing; however, the evidence is lacking on non‐pharmacological behavioral interventions to support cognitive health and diabetes management for individuals with both conditions. The objective of this scoping review was to systematically map the research done in this area, characterize the interventions and outcomes that are under investigation, and identify any existing gaps in knowledge.

**Method:**

Literature searches were conducted electronically using MEDLINE (Ovid), CINAHL (EBSCO), Web of Science Core Collection, PsycINFO (Ovid), EMBASE (Ovid), and AgeLine (EBSCO), limiting to articles published in English, but without any restrictions on publication date. The review protocol was registered prospectively with the Open Science Framework (https://osf.io/2w3sh). Two reviewers independently screened the title, abstracts, and full text articles, respectively.

**Results:**

Database and citation searching yielded 26,532 records. Duplicates were removed, leaving 16740 unique titles and abstracts to be screened for eligibility. Following full text retrieval, 101 potentially relevant papers were screened against the inclusion criteria, leaving 14 articles that were eligible for inclusion in the review (Figure 1. PRISMA). Among the 14 included studies, the non‐pharmacological interventions included diabetes education, Tai Chi, diet and/or exercise, cognitive training, and technology. Three studies showed improved HbA1c, 4 of them showed improved cognitive function, 2 of them showed improved adherence to dietary intake, and 2 reported improved self‐efficacy scores.

**Conclusions:**

Our review indicated heterogeneous non‐pharmacological interventions for adults with comorbid cognitive impairment and diabetes, which demonstrated mixed effects on cognition and diabetes control. Limited interventions have involved family members and been tailored to the needs of both older adults with cognitive impairment and their care partners. The findings indicated the need for developing effective interventions to overcome dual care challenges for older adults with comorbid cognitive impairment and diabetes and their care partners.